# HDAC-an important target for improving tumor radiotherapy resistance

**DOI:** 10.3389/fonc.2023.1193637

**Published:** 2023-07-12

**Authors:** Rui Ling, Jingzhi Wang, Yuan Fang, Yunpeng Yu, Yuting Su, Wen Sun, Xiaoqin Li, Xiang Tang

**Affiliations:** ^1^ Department of Oncology, Affiliated Hospital of Jiangsu University, Zhenjiang, China; ^2^ Department of Radiotherapy Oncology, Affiliated Yancheng First Hospital of Nanjing University Medical School, First People’s Hospital of Yancheng, Yancheng, China

**Keywords:** radiotherapy resistance, HDACs, HDAC inhibitors, DSBR, cell cycle

## Abstract

Radiotherapy is an important means of tumor treatment, but radiotherapy resistance has been a difficult problem in the comprehensive treatment of clinical tumors. The mechanisms of radiotherapy resistance include the repair of sublethal damage and potentially lethal damage of tumor cells, cell repopulation, cell cycle redistribution, and reoxygenation. These processes are closely related to the regulation of epigenetic modifications. Histone deacetylases (HDACs), as important regulators of the epigenetic structure of cancer, are widely involved in the formation of tumor radiotherapy resistance by participating in DNA damage repair, cell cycle regulation, cell apoptosis, and other mechanisms. Although the important role of HDACs and their related inhibitors in tumor therapy has been reviewed, the relationship between HDACs and radiotherapy has not been systematically studied. This article systematically expounds for the first time the specific mechanism by which HDACs promote tumor radiotherapy resistance *in vivo* and *in vitro* and the clinical application prospects of HDAC inhibitors, aiming to provide a reference for HDAC-related drug development and guide the future research direction of HDAC inhibitors that improve tumor radiotherapy resistance.

## 1 Introduction

Radiation therapy is one of the important treatment methods for tumors. It is used as a radical treatment for some tumors and important part of comprehensive tumor treatment. Radiotherapy reduces the risk of local tumor recurrence or kills residual tumor (1, 2). Unfortunately, radioresistance often occurs, leading to the failure of radiotherapy and the progression of patients’ conditions. Research into clinical radiobiology has proposed four biological factors that affect radiosensitivity during radiotherapy: sublethal and potentially lethal injury and repair, cell repopulation, cell cycle redistribution, and reoxygenation (3–5). The occurrence of radiotherapy resistance is an extremely complex process, involving many aspects, such as cell cycle arrest, gene alteration, and differentiation of tumor stem cells, and some external factors, such as stress, hypoxia, and changes in the tumor microenvironment (6, 7). In addition, epigenetic modification plays a role in radiotherapy resistance. Therefore, many scholars and clinical experts have shown great interest in finding targets that improve radiotherapy resistance through the mechanisms of epigenetic changes.

Epigenetic changes refer to changes that are inherited but do not change the DNA genetic code, including DNA methylation and histone modification. Several histone post-translational modifications have been identified, including ubiquitination, phosphorylation, methylation, and acetylation. These modifications can have a profound impact on the interaction between DNA and histones, thereby affecting gene transcription patterns, DNA repair (or replication), and chromosome organization, thereby causing a series of biological changes (8, 9). The acetylation and deacetylation of histone lysine residues are the most powerful modes of epigenetic modification and induced by the histone acetyltransferase (HAT) and histone deacetylase (HDAC) families, which maintain the dynamic balance of histone acetylation or deacetylation modification. Histone acetylation is a dynamic process, and the half-lives of histone acetyl groups are measured in minutes (10). HATs mainly promote the acetylation of histone H3 at lysine-9 and lysine-14, and H4 at lysine-5, lysine-8, lysine-12, and lysine-16 sites, increasing the activities of transcription factors and thus activating gene transcription. HDACs enable the histone N-ϵ-deacetylation of lysine residues to increase the strength of ionic interaction between the positively charged histone tail and negatively charged DNA, causing the end to shift from the nucleosome, making the chromatin structure compact, and finally inhibiting the transcription process (11, 12). Notably, apart from acting as histones, HATs and HDACs are involved in cell cycle progression, differentiation, and apoptosis by activating a variety of non-histones (such as p53 and E2F) (13, 14). Once the balance of between histone acetylation and deacetylation is disrupted, the transcription levels of some specific genes change Substantially and this effect promotes the occurrence and progression of various diseases, including tumors, and tumor chemotherapy and radiotherapy resistance. Therefore, HDACs may be important potential targets for radiosensitization and radioresistance. This article reviews the unique role of HDACs family in tumor radiotherapy resistance and the preliminary achievements and prospects of research into related inhibitors in clinical application.

## 2 Overview of HADCs

### 2.1 Classification of HDACs

A total of 18 members of the HDAC superfamily have been discovered. They can be divided into four categories according to the sizes and spatial structures of these proteins, the functions of these protein, the number of active sites, the subcellular positioning of cells, and variations in the homology of yeast deacetase (15). Class I is the most important HDAC type, including HDAC1, HDAC2, HDAC3, and HDAC8, which are homologous to yeast RPD3. The substrate includes all histones, but the main role is to remove acetyl groups at the 12th site of histone H3. HDAC1, HDAC2, and HDAC8 are only present in the nucleus, and HDAC3 is present in the nucleus and the cytoplasm. Class I HDACs can only co-locate in the nucleus (16). Class II HDACs (HDAC4, HDAC5, HDAC6, HDAC7, HDAC9, and HDAC10) have domains similar to the domain of HDA1, another deacetylase found in yeast. HDAC 4/5/7/9 are included in class IIa HDACs, which rely on calmodulin-dependent protein kinase (CaMK) to continuously shuttle in the cytoplasm and nucleus, and the shuttling movement of HDAC5 may be regulated by the NES domain (17). HDAC6/10 is mainly located in the cytoplasm, known as class IIb HDACs (18). Related studies on the mechanism of their shuttle movement are few. Class III HDACs are nicotinamide adenine dinucleotide (NAD+)-dependent deacetylases, including seven members of the sirtuin (SIRT) family, which are homologous with yeast SIR2, can sense various changes in the nucleus, cytoplasm, and mitochondria. This type of enzymes induces the deacetylation and modification of target proteins (including non-histone substrates) (19). Class IV HDACs include only HDAC 11, which was discovered in 2002, is mainly localized in the nucleus, and may exist in protein complexes containing HDAC6 (20). In contrast to class III HDACs, which rely on NAD^+^, HDAC classes I, II, and IV require zinc as a cofactor (21). Transcription factor transcription factor 1(TCF1) and lymphoid enhancer-binding factor 1 (LEF1) show the intrinsic activities of classes I and III HDACs and have similar structural domains (22). NOC2-like nuclear associated transcriptional repressor (NOC2L) regulates histone acetylation in an independent manner, similar to HDACs (23) (**Table 1**).

**Table 1:** Classification of Histone deacetylases (HDACs) according to the homology with yeast, cellular localization, functions, and their dependent enzyme.

Class	Name	Yeast	Location	Functions	Dependent
Class I	HDAC 1 HDAC 2 HDAC 3 HDAC 8	RPD3	Nuclear	Deacetylate all histones	Zn^2+^
Class IIa	HDAC 4 HDAC 5 HDAC 7 HDAC 9	HDA1	Nuclear/cytoplasmic	Deacetylate nuclear histones	Zn^2+^
Class IIb	HDAC 6 HDAC 10	HDA1	Nuclear/cytoplasmic	Deacetylate nuclear histones Deacetylate non-histones	Zn^2+^
Class III	SIRT 1-7	SIR2	Nuclear/cytoplasmic	Deacetylate mitochondrial Histones Deacetylate non-histones	NAD^+^
Class IV	HDAC 11	Unknow	Nuclear	Unknow	Zn^2+^
Analogues	TCF 1 LEF 1 NOC 2L	Unknown	Nuclear	Deacetylate histones	Unknown

### 2.2 Functions and activities of HDACs

The main function of HDACs is to regulate the balance between the acetylation and deacetylation of substrates, including histone or non-histone substrates. the disruption of this balance can affect the activation or inhibition of various cellular pathways, including JAK/STAT, Wnt/β-catenin, PI3K/AKT/mTOR, MAPK, and NF-kB signaling pathway and other signaling pathways playing important roles in cell cycle regulation, autophagy, apoptosis, DNA damage repair, inflammatory response, and oxidative stress (24–28). For example, in patients with acute leukemia, the inhibition of HDACs can lead to the significant down-regulation of AKT and mTOR, thereby promoting the apoptosis of tumor cells (including the classical and mitochondrial apoptosis pathways) (29). In patients with arterial valve calcification, class I HDACs can up-regulate the activity of the Wnt/β-catenin pathway, leading to the massive proliferation of interstitial cells and sterile inflammation (inflammation caused by non-microorganisms) and the progression of valvular calcification (30). In melanoma, HDAC3 can enhance the tolerance of tumor cells to reactive oxygen species (ROS) and the repair ability of DNA damage by promoting the activation of the MAPK signal pathway (31). HDACs can directly regulate the transcription and expression of cell cycle–related proteins P21, P27, and P53 through chromatin remodeling, thereby affecting changes in the cell cycle. These functions have been confirmed in glioblastoma, gastric cancer, lung cancer, and other tumors (32–35). In addition, HDAC6 and HDAC10 can promote DNA damage repair in tumor cells, such as prostate cancer and neuroblastoma, by directly deacetylating heat shock proteins HSP-27, HSP-70, and HSP-90 (36, 37). HDACs can inhibit non-acetylated modifications, such as the ubiquitination of non-histone substrates (TLR and MYD8) and exert a series of biological effects (38–40). Moreover, HDACs are related to signaling pathways, such as IFN, but the related mechanism still needs further study (41).

The activities of HDACs are regulated by many factors. Inositol pyrophosphates can phosphorylate the target of rapamycin complex 1 (TORC1) and Sch9-dependent transcription factors, facilitate the recruitment of the HDACs of class I to ribosomal and protein synthesis genes, enhance the activity of HDACs, and induce the up-regulation of the HDACs of class I through the interaction with the zinc finger motif in the Sin3-related protein 30 (SAP30) subunit (42). Class IIa HDACs (HDAC4/5/7/9) are phosphorylated by microtubule affinity-regulating kinase 2 (MARK2) at S246, S467, and S632 (coded HDAC4), which promotes the cytoplasmic retention of HDACs and inhibits their deacetylation or transcriptional repression (43). Nuclear actin can combine with HDAC1 and HDAC2 to increase the level of their activity (43). In addition, the activity of HDACs is regulated by microRNA, such as miR-222, which negatively regulates HDAC4, and miR-30c, which negatively regulates HDAC9 (44, 45).

## 3 HDACs and radiotherapy resistance

Radiation therapy is a local treatment method that uses radiation to treat tumors and is one of the three major treatment methods for tumors (46). Radiation includes α, β, and γ rays produced by radioactive isotopes and X-rays, electron beams, proton beams, and other particle beams produced by various X-ray therapy machines or accelerators (47, 48). According to the difference in linear energy transfer (LET), rays are further divided into High-LET and Low-LET clinically. High-energy radiation can directly destroy the single or double strands of DNA in tumor cells, directly leading to the death of tumor cells, whereas low-energy radiation can indirectly cause the death of tumor cells through the generation of oxygen free radicals in cells through ionizing radiation and is affected by the oxygenation of tumor cells, the apoptosis ability of tumor cells, and the cycle of tumor cells (49, 50). Therefore, the DNA damage repair ability of tumor cells, the distribution of cell cycle, and the apoptosis pathway of cells are all factors that lead to tumor radiotherapy resistance, and HDACs are involved in these processes to varying degrees. Furthermore, this review will focus on HDACs and the influences of their biological functions in tumor radiotherapy resistance.

### 3.1 HDACs participate in tumor radiotherapy resistance by affecting DNA SSBR and DSBR

Ionizing radiation can cause DNA single-strand or double-strand breaks (SSBs and DSBs, respectively), which mainly cause radiotherapy-induced tumor cell death (DSBs are more important) (51). Once intracellular DNA breaks, tumor cells repair broken single or double strands through a specific mechanism, and this feature ensures their survival. This process is called SSBR (for SSBs) and DSBR (for DSBs) (52). Once an SSB occurs, it is instantly recognized by poly (ADP-ribose) polymerase 1 (PARP1), which in turn activates recombinant apurinic/apyrimidinic endonuclease 1 (APE1), polynucleotide kinase 3′-phosphatase (PNKP), and other enzymes (53, 54). Finally, recombinant ATP-dependent DNA ligase I (LIG1) connects the broken ends of single strands. DSBR mainly involves homologous recombination (HR) and non-homologous end joining (NHEJ) (55). The HR pathway is initiated by the MRN complex (Mre11-Rad50-NBS1), which recruits ataxia-telangiectasia mutated (ATM) and TIP60 to the DSB site after detecting DSBs, activates ATM, and phosphorylates a series of proteins including histones (H2AX). H2AX is ubiquitinated and finally recruited to breast cancer 1 (BRAC1) to connect with its corresponding site (56). The MRN complex and transcription factor CtBP-interacting protein promote DNA end cleavage, degrading 5′ end DNA and producing 3′ single-stranded DNA (ssDNA). The 3′ ssDNA is coated by ATM-mediated replication protein A (RPA), which protects it from nuclease degradation and removes secondary structures. RPA is replaced by the recombinase RAD51 through a process mediated by ATM, and RAD51 mediates intrusion into the DNA double-stranded template and completes the repair process (57). The ATM-mediated activation of RPA and RAD51 depends on check-point kinase 1 (CHK1) and check-point kinase 2 (CHK2) to amplify the signaling cascade reaction and gain enough time for DSBR (58). The NHEJ pathway recognizes DSBs by Ku70/Ku80 protein dimer and forms a Ku-DNA complex. DNA-dependent protein kinase catalytic subunit (DNA-PKcs) is recruited through 53BP1, and its kinase activity is activated and finish NHEJ pathway.

**Figure 1:**
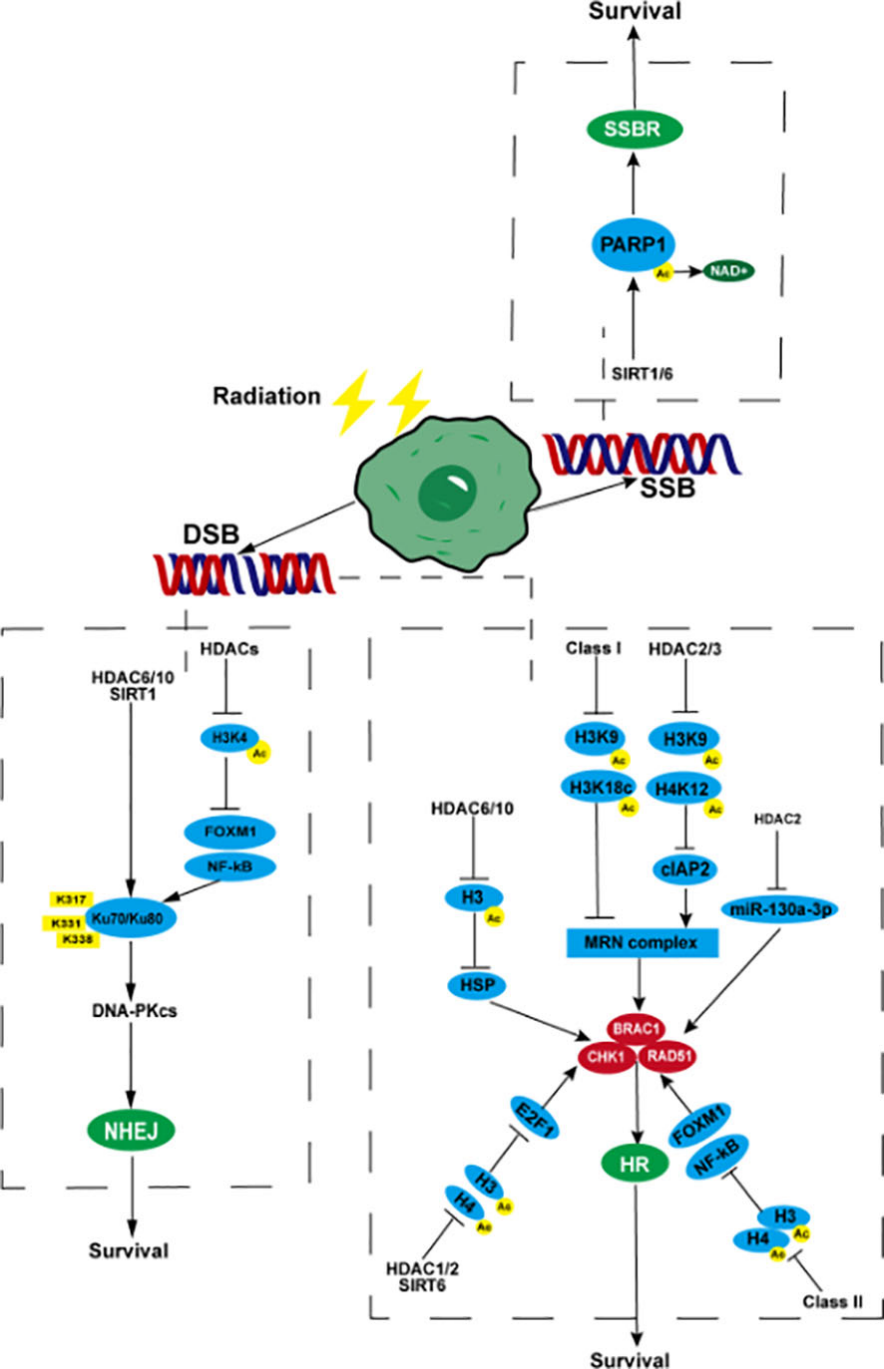
HDACs participate in tumor radiotherapy resistance by affecting DNA SSBR and DSBR. When radiotherapy causes DNA SSBs in tumor cells, SIRT1/6 promotes the acetyl group of PARP1 to move towards NAD^+,^ causing PARP1 to deacetylate and generate activity, promoting SSBR, which is beneficial to tumor cell survival. After DNA double strand breaks, (NHEJ) HDACs promote the binding of NF-kB and FOXM1 to the promoter regions of Ku70/Ku80 through deacetylation of histone H3, enhancing their transcription and expression. HDAC6, HDAC10, and SIRT1 can target deacetylated Ku70 protein lysine residues K317, K331, and K338, enhance their affinity for DNA binding, and initiate the NHEJ pathway. (HR) Class I HDACs reduce the acetylation level of H3K9, H3K18c, and promote the expression of MRN complexes. HDAC2/3 can also deacetylate H3K9, H4K12, and upregulate MRN by activating cIAP2. MRN complexes promote the expression and activity of BRAC1, CHK1, and RAD51, enhancing HR. HDAC6/10 inhibits H3 acetylation, promotes the expression of CHK1 and RAD51 by upregulating HSP, inhibits H3 and H4 acetylation by HDAC1/2 and SIRT6, activates the transcription promoting activity of E2F1, promotes the transcription of BRAC1, CHK1, and RAD51, deacetylates H3 and H4 by class II HDACs, activates NF-kB and FOXM1, and promotes the expression of BRAC1, CHK1, and RAD51. HDAC2 can promote the expression of RAD51 by inhibiting the transcription of miR-130a-3p. PARP1, poly (ADP-ribose) polymerase 1; NHEJ, non-homologous end joining; FOXM1, fork-head box M1; HR, homologous recombination; MRN, Mre11-Rad50-NBS1; cIAP2, cellular inhibitor of apoptosis protein 2; CHK1, checkpoint kinase1; BRAC1, breast cancer 1; SSB, single-strand breaks; DSB, double-strand breaks; SSBR, single-strand break repair; DSBR, double-strand break repair.

SSBR is one of the important reasons for the survival of tumor cells under radiotherapy. PARP1 is involved in the initial stages of SSBR and drives the cells to initiate SSBR. HDACs can promote tumor radiotherapy resistance by affecting the transcriptional expression and activity of PARP1. As early as 2013, Hehlgans found that the HDAC inhibitor (HDACi) NDACI054 used in treating lung, colon, pancreatic, and head and neck cancer has significant cytotoxicity and radiosensitization effects (59). Moert reported that the radiosensitization effect may be related to the down-regulation of PARP1 caused by the inhibition of HDACs activity, thereby inhibiting SSBR in tumor cells after radiotherapy (60). The HDACi-induced down-regulation of PARP1 has been found in breast cancer, prostate cancer, glioblastoma, and other tumors (61–63). The down-regulation or inactivation of PARP1 caused by HDACi may be related to the inhibition of SIRT1 and SIRT6 activities of class III HDACs. SIRT1 and SIRT6 can transfer the acetyl group of PARP1 to the ADP ribose part of NAD+, thereby deacetylating it and activating PARP1 (64) (**Figure 1**). In addition, HDACi enhances the transcription and expression levels not only of the homologous recombinant repair protein PARP1 but also of glutathione (GSH), thereby enhancing the resistance of prostate cancer cells to oxidative stress–induced DNA damage. Oxidative stress is one of the mechanisms underlying ionizing radiation (IR) to kill tumor cells (63). Increase in GSH level depends on class I HDACs HDAC1, HDAC2, HDAC3, and HDAC8, which participate in the regulation of GSH by increasing acetylated histones H3K9 and H3K27 (65, 66). Zacharioudakis found that HDAC1 can inhibit PCNA single ubiquitination (this effect is heavily dependent on the presence of RAD18), maintain PCNA activity, and ultimately enhance the DNA damage of colon cancer HCT-116, osteosarcoma U2OS, and ovarian cancer A2780 cell’s ability to repair (67). This phenomenon exists in gliomas, which is one of the reasons why gliomas are resistant to temozolomide (68). However, whether this mechanism can affect the resistance of tumor cells to radiotherapy remains to be further explored.

DSBs, a highly deleterious DNA damage, occupy important position in radiotherapy-mediated tumor cell death. DSBR is a more important mechanism of tumor radiotherapy resistance. After the formation of DSBs, HDACs deacetylate core histones near the broken DNA strands to compress the chromatin structure and prevent the broken DNA ends from separating from each other, thereby ensuring efficient repair (69). HDACs can promote the repair of DSBs by activating related genes in the HR and NHEJ pathways, eventually leading to the occurrence of radiotherapy resistance. Studies on human squamous cell carcinoma, triple negative breast cancer, prostate cancer, small cell lung cancer, non-small cell lung cancer (NSCLC), hepatocellular carcinoma, and other tumors have shown that HDACs can promote the expression of key proteins in the HR pathway, such as RAD51, CHK1, and BRAC1 (70–75). Transcription and expression can enhance DSBR, thereby inhibiting the radiosensitivity of tumor cells. The regulation of the above proteins may be achieved through the following pathways. In the first pathway, HDACs promote the expression of HSP70 by deacetylating histone H3 (without affecting the deacetylation of histone H4), up-regulating mutp53 expression, and maintaining mutp53 stability, thereby up-regulating RAD51 and CHK1 (75). In addition, class IIb HDACs HDAC6 and HDAC10 are closely related to the regulation of heat shock proteins (76). The second pathway inhibits the acetylation of histone H3 or H4 and promotes the combination of recombinant E2F transcription factor 1 (E2F1) and RAD51, CHK1, and BRAC1 promoters, thereby enhancing the transcription of the above genes (77). Class I HDACs HDAC1 and HDAC2, class III HDACs, SIRT6 are closely related to E2F1 (78, 79). In the third pathway, HDACs can deacetylate histones H3 and H4, facilitating the activation of NF-kB and fork-head box M1 (FOXM1) activated after radiotherapy to combine with RAD51, CHK1, and BRAC1 promoters to promote their transcription and expression (80). Class II HDACs were confirmed to be related to the activity of NF-kB (81). Finally, HDAC2 can promote the expression of RAD51 by inhibiting the transcription of miR-130a-3p (82). However, the impact of HDACs on the MRN complex has been rarely reported. It is currently known that non-small cell carcinoma radiotherapy can cause up-regulation of class I HDACs and lead to a decrease in the acetylation level of histone H3 (such as H3K9, H3K18c, etc.). and higher levels of MRE11-RAD50-NBS1 (MRN) complex expression, thereby causing radiotherapy resistance (74, 83, 84). Expression of the cellular inhibitor of apoptosis protein 2 (cIAP2) can be up-regulated using HDAC inhibitors. As a post-transcriptional regulator of MRE11, cIAP2 down-regulates the level of MRE11 and promotes the ubiquitination and degradation of MRE11, making it lack nuclease activity and weaken the ability to bind to DNA, and improve the radioresistance caused by the up-regulation of HDACs after bladder cancer radiotherapy (85). The up-regulation of cIAP2 may be related to the acetylation of H3K9 and H4K12 after the inhibition of HDAC2 and HDAC3, and the activation of their transcription, but not to HDAC6 (86). HDACs can also participate in tumor radiotherapy resistance by regulating the expression level and activity of key proteins in the NHEJ pathway such as Ku70/Ku80, DNA-PKcs, and 53BP1. Studies in human sarcoma, lymphoma, melanoma, prostate cancer and other tumors have shown that HDACs can promote the expression of ku70 and ku80 proteins and DNA-PKcs related to the NHEJ pathway by acetylating histone H3K4, and enhance tumor cell survival after radiotherapy (87, 88). Studies on high-grade glioma in children have shown that HDACs may promote the binding of NF-kB and FOXM1 to the promoter regions of Ku70/Ku80 and 53BP1 by deacetylating histone H3, thereby enhancing their transcription and expression (80). Studies in lymphoma and prostate cancer have shown that HDAC6, HDAC10, and SIRT1 can target the lysine residues K317, K331, and K338 of the deacetylated Ku70 protein, enhance their binding affinity to DNA to initiate NHEJ, and reduce the lethal effect of radiotherapy and chemotherapy on tumors (87, 89–91) (**Figure 1**).

### 3.2 HDACs participate in tumor radiotherapy resistance by affecting cell cycle regulation

After radiotherapy causes DNA damage in tumor cells, tumor cells not only activate SSBR and DSBR pathways to survive, and block cell cycle checkpoints, resulting in cell cycle arrest. In theory, this can inhibit the cycle proliferation of tumor cells but also provide enough time for DNA damage repair. Once repair is completed, tumor cells can resume their proliferative ability again and leads to the occurrence of radiotherapy resistance (92, 93). Cells in different cycles have different levels of sensitivity to radiotherapy. In theory, tumor cells at the G2/M phase are the most sensitive to radiotherapy. Tumor cells are more likely to be killed and unable to complete HR repair. Relatively insensitive, the cells can easily complete NHEJ and HR repair and survive (93, 94). Therefore, cell cycle redistribution is one of the causes of radiotherapy resistance. The division and proliferation of eukaryotic cells can be divided into four cycles, namely, G1, S, G2, and M phases, which correspond to the early, middle, and final stages of DNA synthesis and the mitosis/meiosis stage. After receiving the mitosis/meiosis signal in the G1 phase, by up-regulating cyclin D, cells activate cyclin-dependent kinase 4/cyclin-dependent kinase 6 (CDK4/CDK6), and form cyclin D-CDK4/6 complex, which make retinoblastoma protein phosphorylation and inactivation, finally lead to the activation of E2F transcription factors and subsequent up-regulation of cyclin E, which binds to and activates cyclin-dependent kinase 2 (CDK2) (94). After triggering DNA replication, the cyclin E-CDK2 complex, which is replaced by cyclin A-CDK2, which activates cyclin B-CDK1 and mediated by cyclin B-CDK1, finally completes cytoskeleton reorganization, chromosome condensation, and mitotic spindle body assembly (95). Moreover, during the above process, CDKs are regulated by various molecular pathways and checkpoints, such as P15, P16, P18, and P19, negatively regulate cyclin D-CDK4/6. P21, P27, and P57 positively regulate cyclin D-CDK4/6 and negatively regulate CDK2 and CDK1. WEE1 negatively regulates CDK1. p53 plays a key role in the cell cycle by positively regulating p21. In addition, E3 ligase complexes (including CDC20, CDC25, and CDH1) can lead to multiple key target proteins in the division cycle ubiquitination degradation (96, 97).

**Figure 2:**
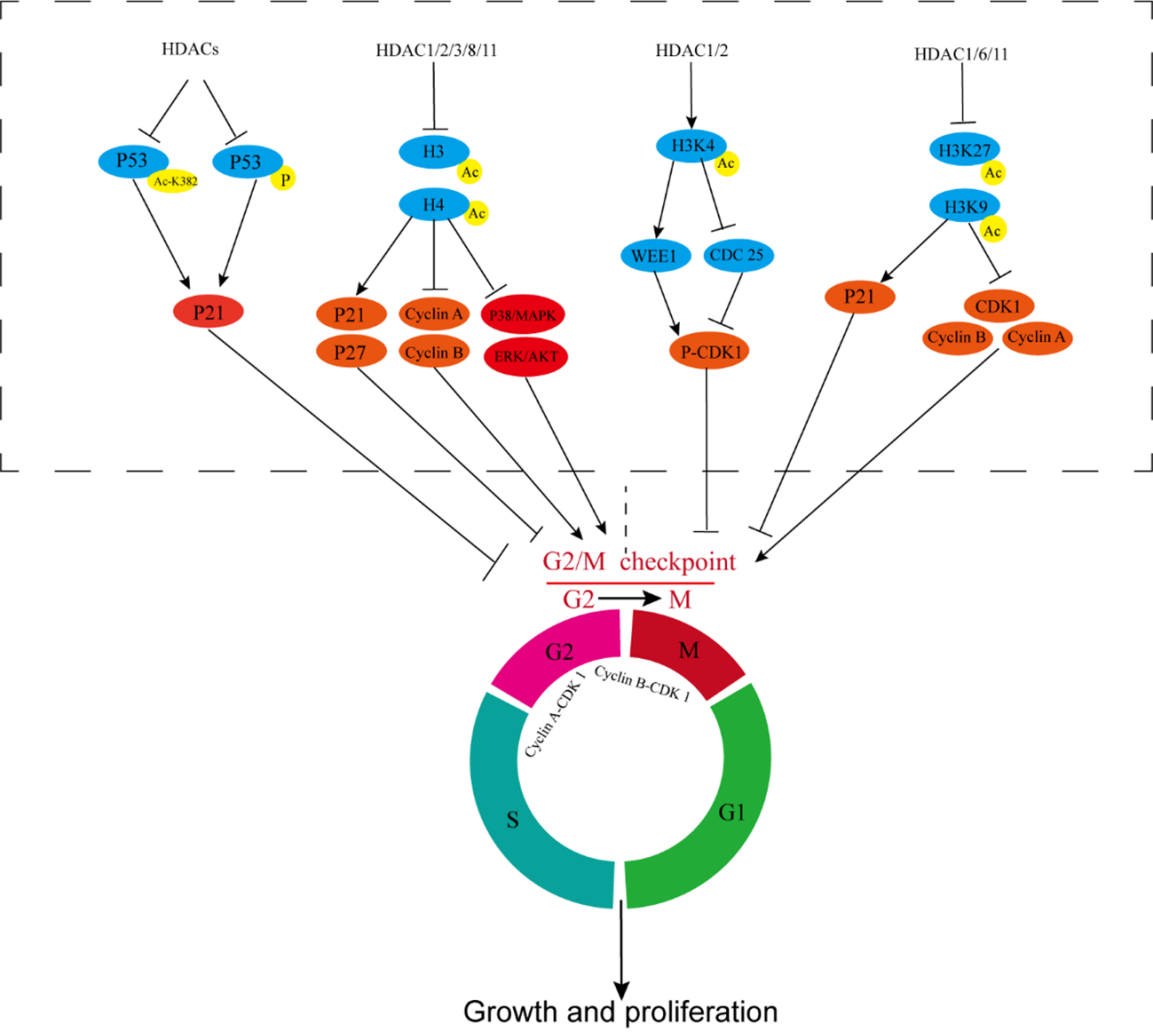
HDACs participate in tumor radiotherapy resistance by affecting cell cycle regulation. HDACs inhibit P53 acetylation and phosphorylation, prevent P53 activation, inhibit P21, and reduce G2/M cycle arrest of cells. HDAC1/2/3/8/11 deacetylates histones H3 and H4, inhibits P21 and P27, upregulates cyclin-A and cyclin-B, and activates ERK/AKT and P38/MAPK, reducing G2/M cycle arrest of cells. HDAC1/2 deacetylates histone H3K4, thereby inhibiting WEE1 and promoting CDC25 transcription, reducing CDK1 phosphorylation, and activating cyclin B-CDK1 activity. HDAC1/6/11 increases the transcription and expression of CDK1, cyclin A, and cyclin B and inhibits the transcription of P21 by inhibiting the acetylation of H3K27 and H3K9. CDC25, Cell Division Cycle Protein 25; CDK1, cyclin-dependent kinase 1.

HDACi plays a considerable role in cell cycle regulation, induces G2/M phase arrest in bladder, breast cancer, and pancreatic cancer; glioblastoma; prostate cancer; leukemia; myeloma; NSCLC; and other tumors, and increases the percentage of G2/M phase cells (98–105). In bladder cancer, HDAC1 and HDAC2 deacetylate histone H3K4, inhibit WEE1, promote the transcription of CDC25, reduce the phosphorylation of CDK1, activate the activity of cyclin B-CDK1, and promote the progress of the G2/M phase (**Figure 2**). These effects promote cell proliferation, reduce the proportion of G2/M cells, and lead to radiotherapy resistance (105). In breast cancer, HDAC1, HDAC6, and HDAC11 can increase the transcription and expression levels of CDK1, cyclin-A, and cyclin-B by the deacetylation of H3K27 and H3K9, can inhibit the transcription of P21, and promote G2/M transformation by inhibiting the acetylation of histones in the specificity protein 1 (SP1)/specificity protein 3 (SP3) regions of P21. These effects reduce the proportion of G2/M cells and sensitivity to radiotherapy (100, 106–108).

Moreover, HDAC6 and SIRT1 can promote mitotic proliferation and reduce the efficacy of radiotherapy by promoting microtubule repolymerization (109, 110). Similar mechanisms exist in cervical cancer, osteosarcoma, and kidney cancer (111, 112). In glioblastoma, the expression levels of HDAC1, HDAC2, HDAC3, and HDAC6 increase in high-grade malignant glioblastoma and are associated with poor prognosis, chemotherapy resistance, and radiotherapy resistance (103, 113). Treatments combining glioblastoma with HDACi and the PARP1 inhibitor olaparib can significantly increase the ratio of G2/M cells and enhance sensitivity to radiotherapy. These effects may be related to HDAC1-3-induced deacetylation of histone H3 and inhibition of transcription of P21 (63). In NSCLC, HDACi inhibits the activity of HDAC1/2, increases Ac-H4 in the p21 promoter region, promotes the transcription and expression of P21, and enhances the G2/M phase cell aggregation induced by ionizing radiation, which plays a role in radiosensitization effect (114, 115). Combining HDACi with cisplatin and anti-EGFR targeted drugs can synergistically enhance the killing effect of ionizing radiation on NSCLC (74). In rhabdomyosarcoma, pan-HDACi inhibits the expression of HDAC1/2/3/8/11, promotes the down-regulation of cyclin-A and cyclin-B and up-regulation of P21 and P27, and reduces the levels ERK and AKT activities. Increase in the proportion of cells in the G2 phase enhances radiosensitivity (116). In prostate cancer, HDAC 1, 2, 3, and 8 are highly expressed, and class I HDACi can promote the acetylation of histone H3 and H4; down-regulate PCNA, cyclin-A, and cyclin-B; up-regulate p21; activate the P38/MAPK pathway; contribute to the G2/M cycle arrest of cells; inhibit the proliferation of prostate cancer cells; and synergistically enhance the killing effect of radiotherapy (104, 117). In colon cancer, HDACi inhibits the activity of HDACs (the most important types are HDAC1 and HDAC2), increases the acetylation level of histones H3 and H4, and enhances the transcription and expression of p21 and p27, which can significantly improve radiotherapy-induced G2/M phase arrest (118, 119).

P53 is an important tumor suppressor gene and plays a key role in cell cycle regulation, apoptosis, and other processes. HDACi can promote the acetylation of P53 lysine K382 residues and the phosphorylation of P53, up-regulate P21, induce G2/M phase arrest, and exert a radiosensitization effect (120–124) (**Figure 2**). However, the radiosensitization effect of P53 on tumor cells is more evident when apoptosis is regulated, which will be our focus in the subsequent section.

### 3.3 HDACs participate in tumor radiotherapy resistance by affecting apoptosis.

Radiotherapy can cause DNA damage and cycle arrest in tumor cells. During cycle arrest, tumor cells that fail to complete damage repair eventually undergo apoptosis (124). However, some tumor cells can recover their activity and continue to proliferate after radiotherapy and replace cells lost because of radiotherapy-induced apoptosis (125). Therefore, the curative effect of radiotherapy is related to the growth and loss rates of cells, and the proliferation and apoptosis of tumor cells are extremely important to the curative effect of radiotherapy. In apoptosis, also called programmed cell death, apoptotic cells stop growing and dividing and then die. Once cells are damaged and exceed their own repair capacities during chemotherapy or radiotherapy, apoptosis can be initiated (126).

**Figure 3:**
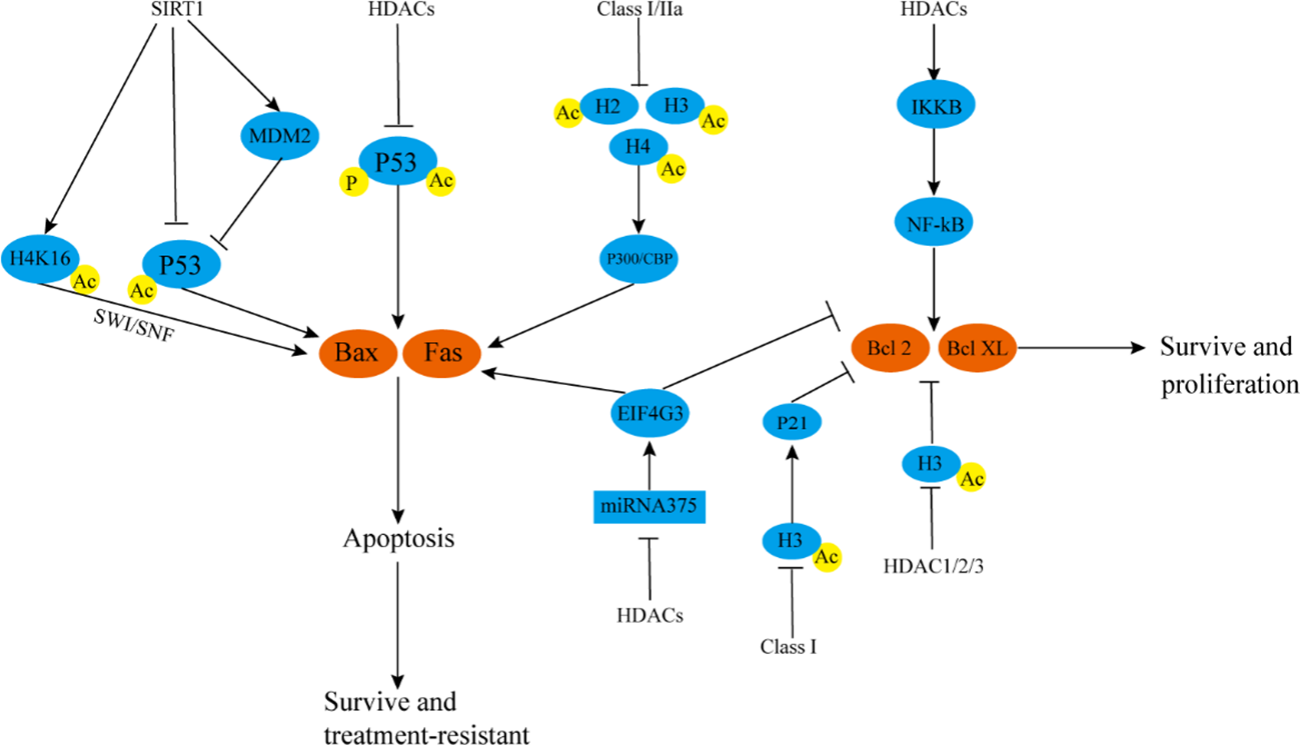
HDACs participate in tumor radiotherapy resistance by affecting autophagy and apoptosis. SIRT1 deacetylates H4K16, thereby inhibiting SWI/SNF, inhibiting Bax and Fas transcription, and reducing cell apoptosis SIRT1 can also directly inhibit P53 acetylation or negatively regulate P53 by up-regulating MDM2, reducing Bax and Fas transcription, and preventing cell apoptosis. HDACs can reduce P53 acetylation and phosphorylation levels, inhibit P53 activity, and negatively regulate Bax and Fas. Class I and IIa HDACs can inhibit acetylation of H2, H3, and H4, reduce P300/CBP activity, and downregulate Bax and Fas. HDACs can also inhibit Bax protein and up-regulate inhibit Bcl-2 protein by inhibiting the activity of miRNA-375/EIF4G3 axis. HDACs can promote IKKB degradation, activate the NF-kB pathway, and promote the expression of Bcl-2/Bcl-XL. Class I HDACs inhibit histone H3 acetylation, down-regulate P21, and positively regulate Bcl-2/Bcl-XL. HDAC1/2/3 promotes the transcription of Bcl-2/Bcl-XL by directly downregulating Ac-H3. These mechanisms are conducive to the survival of tumor cells. SWI/SNF, switch/sucrose nonfermentable; MDM2, mouse double minute 2; EIF4G3, eukaryotic translation initiation factor 4 gamma 3.

HDACs can inhibit apoptosis and promote cell proliferation (127–129). Moreover, they can inhibit the acetylation and phosphorylation of P53, inhibit its activity, and then inhibit the transcription and expression of pro-apoptotic genes, such as *Fas* and *Bax*, thereby inhibiting apoptosis (120) (**Figure 3**). However, P53 often mutates, which not only promotes tumor growth and metastasis but also leads to chemotherapy resistance and radiotherapy resistance. P53 mutation has been reported in glioblastoma. HDACi can promote cell apoptosis and exerts anti-proliferation effects and produces a strong radiosensitization effect. In P53 wild-type glioblastoma, HDACi has no obvious radiosensitization effect (130), which led to subsequent research focusing more on mutp53 tumors. However, with the deepening of relevant research and the emergence of new technologies, in recent years, HDACi has been reported that can also improve drug and radiotherapy resistance in wtP53 tumor cells, although the proapoptotic effect of HDACi in wtP53 tumors is significantly weaker than that in mutP53 tumors (131, 132). However, the proapoptotic effect of HDACi in wtP53 tumors is significantly weaker than that in mutP53 tumors. In human squamous cell carcinoma, HDACi can cooperate with radiotherapy and increase the phosphorylation level of wtP53, promote the expression of Bax protein, and promote cell apoptosis (133). But the effect is significantly weaker than that of mutP53 tumorsHDAC3 can inhibit the acetylation of P53 and promote the signal activity of NF-kB, thereby inhibiting cell cycle arrest and apoptosis (133, 134). In retinoblastoma, HDACi can simultaneously promote the acetylation, phosphorylation, and nuclear relocation of P53, activate P53, up-regulate Bax/Fas, and enhance radiosensitivity (135). In mouse endometrial carcinoma, the selective HDAC SIRT1 inhibitor significantly promotes the degradation of P53 negative regulator, mouse double minute 2, prevents its binding with P53, acetylates P53, enhances the activity of P53, promotes the expression of Fas/Bax, and causes cell apoptosis (136). In addition to the direct deacetylation of P53, SIRT1 can deacetylate histones (the most important is H4K16) and regulate the transcription of Fas and Bax through switch/sucrose nonfermentable (SWI/SNF) (137, 138).

HDACi not only up-regulates pro-apoptotic genes, such as P53/Fas/Bax, but also up-regulates pro-apoptotic proteins, such as Bax and inhibits anti-apoptotic genes, such as Bcl-2/Bcl-XL, independent of P53. In lung cancer and other tumors, after the activities of class I (HDAC1, HDAC2, HDAC3, and HDAC8) and class IIa (HDAC4, HDAC5, and HDAC7) HDACs are inhibited, the levels of acetylation of H2, H3, and H4 increases, and p300/CBP binds with acetylated chromatin at a target protein promoter, promoting the transcription of downstream Bax and other apoptotic proteins (139, 140). In tongue cancer, HDACi can inhibit the phosphorylation and degradation of IKKB, eliminate the activation of NF-kB induced by TNF-α, down-regulate Bcl-2/Bcl-XL, promote the apoptosis of tongue cancer cells, and improve the efficacy of radiotherapy (141). In lung squamous cell carcinoma, HDACi up-regulates Bax and inhibits Bcl-2 expression by activating the miRNA-375/eukaryotic translation initiation factor 4 gamma 3 (EIF4G3) axis, and the effect is further enhanced when HDACi is used in combination with radiotherapy. In glioblastoma, HDACi does not increase the expression level of pro-apoptotic Bax. Rather, it down-regulates the mRNA level of anti-apoptotic Bcl-2/Bcl-XL, and this effect may be related to HDACi-induced inhibition of HDAC1-3 activity and increase in Ac-H3 level (142, 143). In pancreatic cancer, HDACi combined with radiotherapy can up-regulate Bax protein, inhibit Bcl-2 protein expression, promote cell apoptosis together with HR pathway, and improve the efficacy of radiotherapy. Increased level of protein H3 acetylation is related to the promotion of P21 transcription. P21 up-regulates Bax protein and inhibits Bcl-2 protein expression (144, 145) (**Figure 3**). In cervical cancer, the proportion of apoptotic cells can be doubled by the simultaneous use of autophagy inhibitors after the HDACi treatment of cells (146). In glioma and colon cancer, the combined use of HDACi and autophagy inhibitors can significantly increase the level of apoptosis after radiotherapy and enhance the efficacy of radiotherapy (118, 147). Autophagy is a double-edged sword in tumors, but in most cases, autophagy can reduce the rate of apoptosis of tumor cells. However, whether HDACi plays a role in autophagy by inhibiting the activity of HDACs, whether it can directly regulate the autophagy response, and whether there is a synergistic mechanism for enhancing apoptosis after combined use with autophagy inhibitors are unclear. This is a problem that cannot be ignored in the field of radiotherapy research and clinical application of HDACs. The combined use of HDACi and BRD4 inhibitor JQ1 in the treatment of soft-tissue sarcoma and undifferentiated pleomorphic sarcoma cells can significantly inhibit mTOR signaling and promote autophagy (148). These mechanisms possibly underlie autophagy regulation by HDACi and HDACi-induced development of drug resistance.

### 3.4 HDACs participate in tumor radiotherapy resistance in other ways

DNA damage repair, cell cycle regulation, and apoptosis are relatively independent but inter-related processes. For example, cells can undergo cycle arrest when DNA is damaged or replication errors occur. If the damage is difficult to repair, tumor suppressor genes, such as P53, are activated, and damaged cells undergo apoptosis. These factors mainly affect the sensitivity of tumor radiotherapy. Our previous study showed that in some tumors, HDACs can simultaneously affect DNA damage repair, cell cycle regulation, and apoptosis regulation, thereby enhancing tumor radiotherapy resistance. HDACs can also promote tumor radiotherapy resistance and drug resistance by affecting tumor stem cells, tumor immunity, and oxidative stress.

Cancer stem cells (CSCs) constitute a subset of cells in tumors that are in a stem cell state and have stem cell characteristics. They can self-renew and differentiate and may be related to treatment resistance, tumor recurrence, and metastasis (149). In glioblastoma, pan-HDACi can significantly inhibit the activity of CSCs and reduce the expression of box protein-2 (SOX2) and CD44, which are related to CSCs. When combined with the MEK inhibitor trimetinib, pan-HDACi can significantly promote the killing effect of radiotherapy on glioma CSCs and decreases resistance to radiotherapy (113, 150).

**Figure 4:**
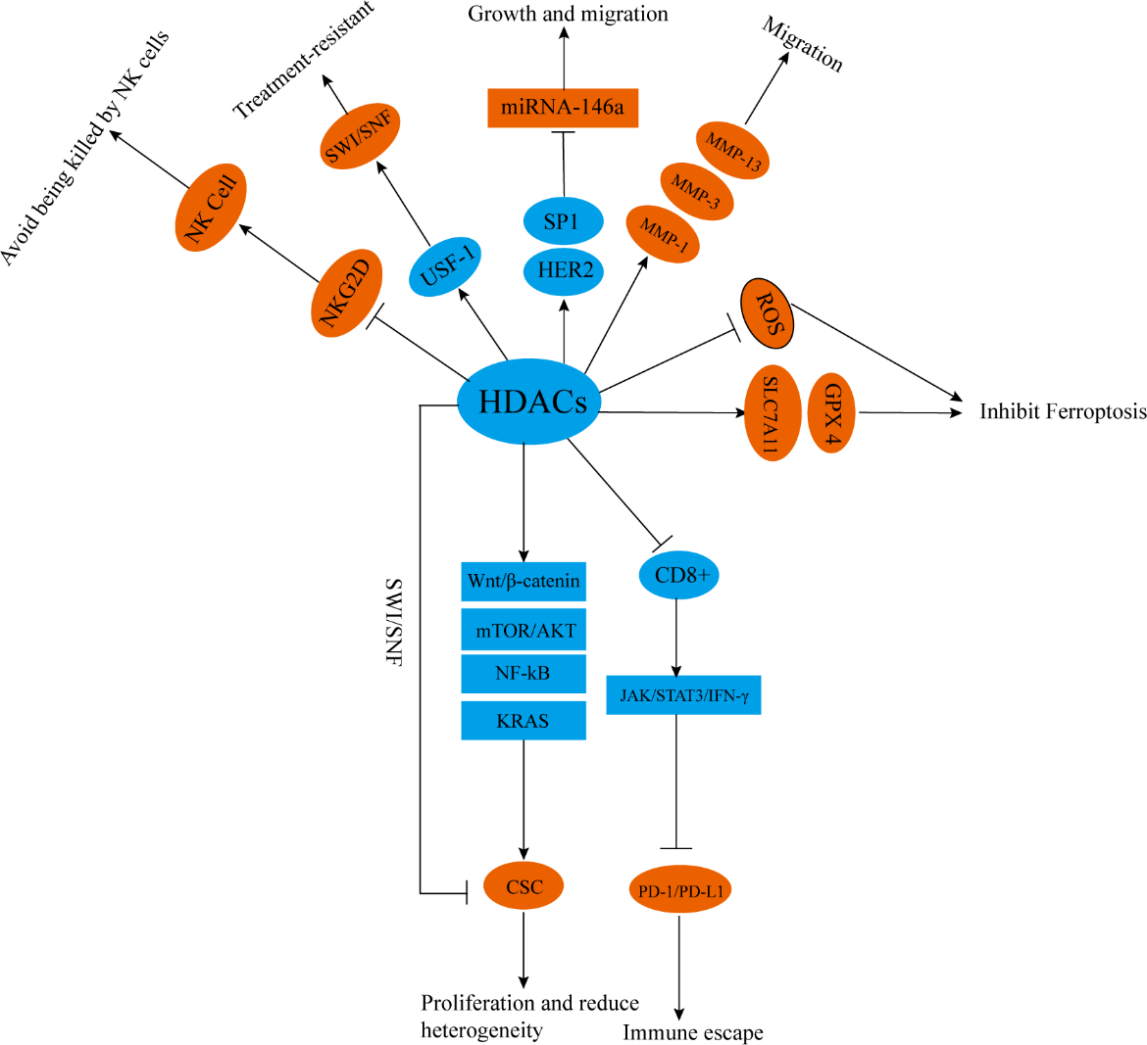
HDACs participate in tumor radiotherapy resistance in other ways. HDACs promote the differentiation and proliferation of CSCs through chromatin remodeling and activating Wnt/β- Catenin, mTOR/Akt, NF-kB, and KRAS signaling pathways. HDACs inhibit the proportion of CD8^+^T cells and reduce the activity of JAK/STAT3/IFN-γ signal pathways, enhancing the expression of PD-1/PD-L1. HDACs can also downregulate the expression of NKG2D, inhibit the activity of NK cells, or enhance SWI/SNF by upregulating USF-1. HDACs can promote the upregulation of MMP-1, MMP-3, and MMP-13 through, upregulate SLC7A11 and GPX4, and enhance HER2/SP1/miR-146a mediated miR-146a silencing. HDACs can also inhibit lipid peroxidation and block ROS mediated signal transduction. NKG2D, NK group 2 member D; SWI/SNF, switch/sucrose nonfermentable; USF-1, upstream stimulatory factor1; MMP-1, matrix metalloproteinase 1; MMP-3, matrix metalloproteinase 3; MMP-13, matrix metalloproteinase 13; SLC7A11, solute carrier family 7, member 11, GPX4; glutathione peroxidase 4, SP1; transcription factor specificity protein 1.

The roles of HDACi in inhibiting CSC proliferation in glioblastoma may be inhibiting the activity of HDAC4, blocking the activation of the Wnt/β-catenin pathway, enhancing MHC class I and adipose most abundant gene transcript protein expression. These activities enhance the cytotoxic effect of T cells on CSCs (151). In prostate cancer, prostate cancer stem cells show less lethal DNA DSBs after radiotherapy, and this feature is one of the reasons for radiotherapy resistance. When HDACi is used to inhibit the activity of HDAC1/3/8, the level of activity is reduced by chromatin remodeling. Heterochromatin levels in prostate cancer CSCs reverse their resistance to radiotherapy (152). In ovarian teratomas and testicular embryonal tumors, a novel inhibitor against HDAC1 has selective antiproliferative effects on cancer stem-like cells and reduces tumor therapy resistance. In colon cancer, the combined use of pan-HDACi and 5-FU can inhibit c-Myc, mTOR/AKT, NF-kB, and KRAS signaling pathways, thereby inhibiting the differentiation and proliferation of CSCs (153) (**Figure 4**).

Combining radiotherapy with immunotherapy improves the efficacy of radiotherapy (154). In mouse models, HDACi increases the proportion of CD8^+^ cells, activates the JAK/STAT3/IFN-γ pathway, and enhances immune activation and radiation sensitivity induced by PD-1 inhibitors (155). However, despite that HDACi enhances the sensitivity of PD-1/PD-L1 inhibitors to radiotherapy, it partially increases the expression level of PD-1/PD-L1, which may affect the efficacy of immunotherapy (156). Therefore, when radiotherapy, immunotherapy, and HDACi are used in combination, how to prevent the activation of the PD-1/PD-L1 pathway remains a problem that limits the clinical application of HDACi.

IR can kill tumor cells by generating free radicals or ROS (157). Tumor cells or tumor stem cells resistant to radiotherapy often maintain low levels of ROS and have strong anti-oxidative stress effects (158). HDACi combined with radiotherapy can significantly up-regulate Ac-H3 and Ac-H4, enhance lipid peroxidation in human melanoma cells, and promote ROS-mediated signal transduction, thus activating death receptors and intrinsic apoptotic pathways (159). The inhibition of HDAC1/2/4/6 can switch adrenocortical carcinoma from an epithelial-like phenotype to a mesenchymal-like phenotype through chromatin remodeling, significantly reducing solute carrier family 7, member 11 (SLC7A11), and glutathione peroxidase 4 (GPX4), which promotes lipid peroxide–mediated ferroptosis (160).

Finally, in breast cancer, the inhibition of HDAC6 can inhibit the growth and proliferation of tumors by reducing the glycolytic ability of breast cancer cells and reduce tumor therapy resistance (161). The inhibition of HDAC1/2/4 can reverse the radiotherapy-induced up-regulation of metalloproteinases matrix metalloproteinase 1 (MMP-1), matrix metalloproteinase 3 (MMP-3), and matrix metalloproteinase 13 (MMP-13) by inhibiting chromatin remodeling and plays a radiosensitizing role (162). HDACi can block the silencing of miR-146a mediated by HER2/SP1/miR-146a, decreases resistance to HER2 targeted therapy, activates the Fas pathway of death receptors, promotes cell apoptosis, and enhances the killing effect of radiotherapy. This mechanism may be related to the increased level of acetylation of histone H3K56 in the miR-146a promoter region after HDAC1 is inhibited (163). HDACi can increase the acetylation of histones H3 and H4, inhibit the binding of upstream stimulating factor 1 (USF-1) to chromatin, thereby inhibiting SWI/SNF and reversing the radiotherapy resistance of human prostate cancer cells (164). HDACi (mainly inhibiting HDAC4) can up-regulate NK group 2 member D ligand and enhance the killing effect of NK cells and the sensitivity of NSCLC and HCC to radiotherapy (165, 166) (**Figure 4**).

## 4 Clinical application of HDAC inhibitors

HDAC inhibitors have made initial breakthroughs in clinical treatment. Seven HDACi have been approved for clinical use: sodium phenylbutyrate (2000 USA), Vorinostat (2006 USA), valproic acid (2008 USA), Romidepsin (2010 USA), Belinostat (2014 USA), Panobinostat (2015 USA), and Chidamide (2015 CHN). Six HDAC inhibitors have been approved by the FDA for the treatment of peripheral T-lymphoma, cutaneous T-cell lymphoma,and multiple myeloma, and one HDAC inhibitor has been approved by the State Drug Administration of China for peripheral T-lymphoma and breast cancer (167–169). The seven HDAC inhibitors can be divided into two categories: non-selective pan-HDAC inhibitors, including Vorinostat, Rromidepsin, and Belinostat, and HDAC selective inhibitors, such as Chidamide, which are effective against HDAC1, HDAC2, HDAC3, and HDAC10 (170, 171). In addition, the selective HDAC inhibitor Entinostat (inhibits HDAC1, HDAC2, and HDAC3), is currently in phase 3 clinical trial (172). Although HDAC selective inhibitors effectively reduce the side effects of pan-HDACi and expands the clinical application, they are mainly used in hematological tumors, and their application to solid tumors is still limited. HDACi often needs to be combined with other targeted drugs in solid tumors to achieve partial efficacy. Therefore, the current drug development direction tends to be dual-target HDAC inhibitors, such as CUDC-101, which can simultaneously inhibit HDAC, EGFR, and HER-2; CUDC-907, which can simultaneously inhibit HDAC/PI3K; and 4SC-202, which can simultaneously inhibit HDAC/LSD1. However, these drugs are currently in phase I or phase II clinical trials (173–175) (**Table 2**).

**Table 2:** The HDAC inhibitors used commonly and their targets and indications.

Name	HDAC	1	2	3	4	5	6	7	8	9	10	11	Sirts	Others	Indication	Phase	References
**Sodium phenylbutyrate**		**✓**	**✓**	**✓**	**✓**	**✓**		**✓**			**✓**			**/**	**UCDS** **CTCL**	**2000**	**(** 176 **)**
**Vorinostat**		**✓**	**✓**	**✓**	**✓**		**✓**	**✓**		**✓**				**/**	**CTCL AML**	**2006**	**(** 177 **)**
**Valproic acid**		**✓**	**✓**	**✓**			**✓**		**✓**	**✓**				**/**	**EP** **CTCL**	**2008**	**(** 178 **)**
**Romidepsin**		**✓**	**✓**		**✓**		**✓**		**✓**		**✓**		**✓**	**/**	**CTCL PTCL**	**2010**	**(** 179 **)**
**Belinostat**		**✓**	**✓**	**✓**	**✓**	**✓**	**✓**	**✓**	**✓**	**✓**			**✓**	**/**	**PTCL**	**2014**	**(** 180 **)**
**Panobinostat**		**✓**	**✓**	**✓**	**✓**		**✓**	**✓**	**✓**	**✓**				**/**	**MM**	**2015**	**(** 181 **)**
**Chidamide**		**✓**	**✓**	**✓**							**✓**			**/**	**PTCL** **BRCA**	**2015**	**(** 182 **)**
**Entinostat**		**✓**	**✓**	**✓**										**/**	**NSCLC** **BRCA**	**III**	**(** 181 **)**
**CUDC-101**		**✓**	**✓**	**✓**	**✓**	**✓**	**✓**	**✓**	**✓**	**✓**	**✓**			**EGFR HER2**	**Pca** **HCC**	**I/II**	**(** 183 **)**
**CUDC-907**		**✓**	**✓**	**✓**	**✓**	**✓**	**✓**	**✓**	**✓**	**✓**	**✓**	**✓**		**PI3K**	**DLBCL**	**II**	**(** 184 **)**
**4SC-202**		**✓**	**✓**	**✓**										**LSD1**	**TOPAS**	**I**	**(** 185 **)**
**PCI-24781**		**✓**					**✓**		**✓**		**✓**		**✓**	**/**	**GBM** **RCC**	**III**	**(** 181 **)**
**MS-275**		**✓**	**✓**	**✓**						**✓**				**/**	**AML** **ALL**	**II**	**(** 181 **)**
**TMP195**					**✓**	**✓**	**✓**	**✓**	**✓**	**✓**				**/**	**BRCA**	**/**	**(** 186 **)**
**Abexinostat**		**✓**	**✓**	**✓**			**✓**		**✓**		**✓**			**/**	**RCC** **DLBCL**	**III**	**(** 187 **)**

/ means inhibitors cannot inhibit substrates other than HDAC, or no relevant clinical drug experiments have been conducted.

✓ means inhibitors can suppress this HDAC subtype.

Clinical research into HDAC inhibitors as radiosensitizers has generated preliminary results. The NCT00455351 study showed that compared with radiotherapy alone, Vorinostat combined with pelvic radiotherapy reduced the volume of pelvic tumors by an average of 26%, and it was well tolerated (183). A clinical study on the treatment of glioma with Vorinostat combined with temozolomide and radiotherapy showed that the OS of patients at 15 months was 55.1%, and the median overall survival period was as high as 16.1 months (184). The phase I clinical study of Vorinostat combined with concurrent radiotherapy and chemotherapy in the treatment of head and neck square cell carcinoma showed that the CR rate of patients receiving this protocol was as high as 96.2%, and the estimated 5 year overall survival rate and 5 year disease-free survival rate were 68.4% and 76.6%, respectively. Tolerance to the adverse effects of radiotherapy was significantly improved (185).

HDACi plays an anti-cancer role mainly by targeting Class I, II and IV HDACs to regulate histone acetylation status, thereby changing the tumor microenvironment, inhibiting the transmission of tumor related signal pathways, inhibiting tumor cell cycle and inducing apoptosis, and enhancing the killing of immune cells to tumors. Due to the high metabolic characteristics of tumor cells, HDACi has a higher uptake concentration in non-solid tumor cells, but the drug concentration in solid tumor tissue is relatively low, possibly due to the anti-angiogenic effect of HDACi, which leads to insufficient blood supply in solid tumor tissue (188, 189). Dual or multi target delivery may help solve the problem of low uptake of HDACi in solid tumors. Compared to traditional anti-tumor drugs, the advantage of HDACi lies in its ability to directly act on the key link of gene abnormal expression. However, there are also disadvantages, such as the high sequence similarity of some HDACs, which makes the target recognition and drug delivery of inhibitors challenging, leading to corresponding adverse reactions. Moreover, HDACi usually uses Zn^2+^ binding groups, including hydroxamic acid, mercaptan, carboxylic acid, ketone or 2-aminoaniline. These functional groups strongly bind with other important metal enzymes to produce corresponding cytotoxicity, thus limiting the clinical application of HDACi.

## 5 Prospects

HDACs widely exist in eukaryotes and play an important role in regulating histone acetylation–deacetylation balance. High HDAC expression levels have been observed in various tumors and are associated with the poor differentiation and prognosis of tumors. A large number of studies have confirmed that HDACi plays an important role in improving tumor drug resistance and radiotherapy resistance. Although HDACi requires a large oral dose in the treatment of tumors, side effects are significantly reduced owing to the development of highly selective HDACi, which has potential clinical applications. HDACi in combination with immunotherapy, targeted therapy, and radiotherapy, and chemotherapy may improve tumor drug resistance, radiotherapy resistance, and immunotherapy insensitivity. In addition, HDACi may reduce some adverse reactions during tumor treatment, such as radiation dermatitis and bone marrow suppression.

HDACi not only improves the sensitivity of tumors to radiotherapy but also acts as a protective agent for normal tissues and increases the maximum dose of normal tissue tolerance to radiotherapy. These features may allow patients to receive higher doses of radiotherapy. However, research supporting this point of view is lacking, although we believe that it is an important direction in the future research of HDACi *in vitro* and *in vivo*. In summary, we believe that HDACi has great potential in the combination therapy of tumors and protection of normal tissues. Drug development targeting HDAC targets may become an important part of future cancer treatment, especially radiosensitive tumor treatment.

## Conflicts of interest

The authors declare that the research was conducted in the absence of any commercial or financial relationships that could be construed as a potential conflict of interest.

## Author contributions

XT, XL and WS provided direction and guidance throughout the preparation of this manuscript. RL and JW wrote and edited the manuscript. YF, YY, YS collected and prepared the related papers. All authors contributed to the article and approved the submitted version.
